# Cross-sectional study of malocclusion in Spanish children

**DOI:** 10.4317/medoral.19096

**Published:** 2013-08-29

**Authors:** José M. Almerich-Silla, José M. Montiel-Company, Carlos Bellot-Arcís, Neus Puertes-Fernández

**Affiliations:** 1 Tenured Lecturer, Stomatology Department, Faculty of Medicine and Dentistry, University of Valencia (Spain); 2 Post-Doctoral Assistant Professor, Stomatology Department, Faculty of Medicine and Dentistry, University of Valencia (Spain); 3 Adjunct Professor, Stomatology Department, Faculty of Medicine and Dentistry, University of Valencia (Spain); 4 PhD student, Stomatology, Department, Faculty of Medicine and Dentistry, University of Valencia (Spain)

## Abstract

Objectives: This study was conducted to determine the orthodontic treatment need of the child population of the Valencia region of Spain, employing the DAI and the IOTN, to examine the relations between treatment need, socio-economic data and gender and to assess the diagnostic agreement between the two indices. Study Design: A cross-sectional descriptive study was conducted in a random representative sample of the schoolchild population of the Valencia region of Spain. The sample size was a total of 765 children aged 12 and 15 years at 39 schools. Results: The orthodontic treatment need assessed by the DAI was 21.7% at 12 years of age and 14.1% at 15 years. The orthodontic treatment need assessed by the IOTN DHC was 20.9% at 12 years of age and 12.7% at 15 years. The diagnostic agreement between the DAI and the modified IOTN was moderate, with Kappa scores of 0.426 at 12 years of age and 0.415 for the 15-year-old group. Conclusions: Approximately 20% of the children needed orthodontic treatment. Neither gender nor social class appeared to exert a significant influence on orthodontic treatment need.

** Key words:**Orthodontics, epidemiology, children, malocclusion.

## Introduction

Many occlusal or orthodontic treatment need indices are described in the literature, although there is no consensus on which it is best to use (1). Of them all, the Dental Aesthetic Index (DAI) and the Index of Orthodontic Treatment Need (IOTN) have demonstrated their validity in epidemiological studies; the DAI is even used for the WHO’s epidemiological surveys, increasing its use and acceptance among the scientific community.

The IOTN classifies malocclusions according to the occlusal components considered important for health and dental aesthetics. Its aim is to identify which individuals would most benefit from orthodontic treatment. The IOTN comprises a Aesthetic Component (AC) with 10 severity levels and a Dental Health Component (DHC) with five severity levels. The two are analysed separately and although they cannot be united into a single score, they can be combined to classify the patient’s orthodontic treatment need as Yes or No. This result is known as modified IOTN.

The distinctive feature of the DAI is that this orthodontic index relates the clinical and aesthetic components mathematically to give a single score. This can be classed into 4 grades of orthodontic treatment need.Both of these indices have been used in previous epidemiological studies to assess orthodontic treatment need in Spain (2,3).

This study was conducted to determine the orthodontic treatment need of the child population of the Valencia region of Spain, employing the DAI and the IOTN, examine the relations between treatment need, socioeconomic data and gender and assess the diagnostic agreement between the two indices.

## Material and Methods

A descriptive cross-sectional study was conducted in a random representative sample of the schoolchild population of the Valencia region of Spain. Cluster sampling was performed to decide the schools in which the examinations would be conducted. All the schools in the Valencia region were included in the sampling process. It was also estimated that the mean number of examinations to be carried out in each of the selected schools should be between 15 and 25 pupils attending a total of 39 schools. The examinations were carried out in a classroom at each school. The examination instruments employed were a WHO-type periodontal probe and a plain mouth mirror. The sample size was 765 children aged 12 and 15 years.

The 3 examiners underwent prior calibration to assess the agreement between their DAI and IOTN orthodontic treatment need diagnoses of study models and those of a reference examiner acting as a ‘gold standard’ (Kappa >0.85). A second calibration test was then carried out with 20 pupils, examined under the same conditions as in the survey to be conducted (Kappa >0.85).

To determine the orthodontic treatment need, the Dental Aesthetic Index (DAI) and both components (DHC and AC) of the Index of Orthodontic Treatment Need (IOTN) were employed. The aesthetic component (AC) was assessed by both the child (AC patient) and the examiner (AC examiner).

The classification of social class was based on that of the UK Registrar (4). Social classes I – professional – and II – intermediate – were considered high. Social classes III N – skilled non-manual – and III M – skilled manual – were considered middle. Social classes IV – partly skilled – and V – unskilled – were considered low. All others were not classified.

The study was approved by the Ethical Committee of the University of Valencia and the Helsinki recommendations for this type of study were followed. Informed consent was obtained from the children’s parents to conduct the examinations, which were performed in November and December 2010.

The data were stored in a Microsoft® Access 2007® database. The statistical analysis was carried out with IBM® SPSS v 19.0® software.

The univariate descriptive statistics obtained were the means and 95% confidence intervals (CI 95%) for the quantitative variables and the percentages and 95% confidence intervals (CI 95%) for the categorical variables. For bivariate data analysis the chi square test was used for comparison of proportions, with a significance level of p≤0.05. The Kappa statistic was employed to determine diagnostic agreement.

## Results

Of the 765 children examined, 397 were 12 years old and 368 were 15 years old. The percentage who had received orthodontic treatment was significantly higher in the 15 year-old age group than among the 12-year-olds: 27.2% for the former compared to 7.1% for the latter (chi-square test p=0.000).

The orthodontic treatment need determined by the DAI was 21.7% in grades 3 and 4 at 12 years of age and 14.1% in grades 3 and 4 at 15 years of age. The mean DAI scores were 26.6 and 24.5 at 12 and 15 years respectively. The 15-year-old group exhibited significantly less treatment need than the 12-year-olds (chi-square test p=0.003).  Table 1 shows the percentage distribution and mean scores in each grade of treatment need according to the DAI for the two age groups.

**Table 1:**
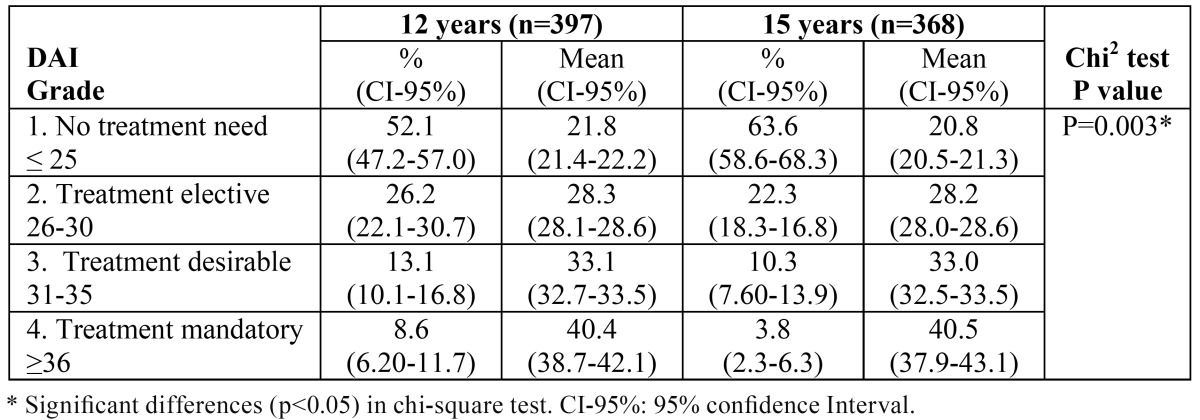
Percentage and mean distribution of Dental Aesthetic Index (DAI) treatment need grades at 12 and 15 years of age.

With the IOTN DHC, the treatment needs obtained were 20.9% in grades 4 and 5 at 12 years of age and 12.7% in grades 4 and 5 at 15 years, showing significant differences (chi-square test p=0.001). With the IOTN AC the differences were equally significant (chi-square test p=0.000), as the results for grades 8-10 were 5.5% for the 12-year-olds and 1.1% for the 15-year-olds as assessed by the children and 7.6% and 3.3% respectively as assessed by the examiners ( Table 2). The need determined by the modified IOTN (IOTN DHC grades 4-5 or patient-assessed IOTN AC grades 8-10) was 23.7% (19.7%-28.1%) at 12 years and 13.3% (10.2%-17.1%) at 15 years, a significant difference (chi-square test p=0.000).

**Table 2:**
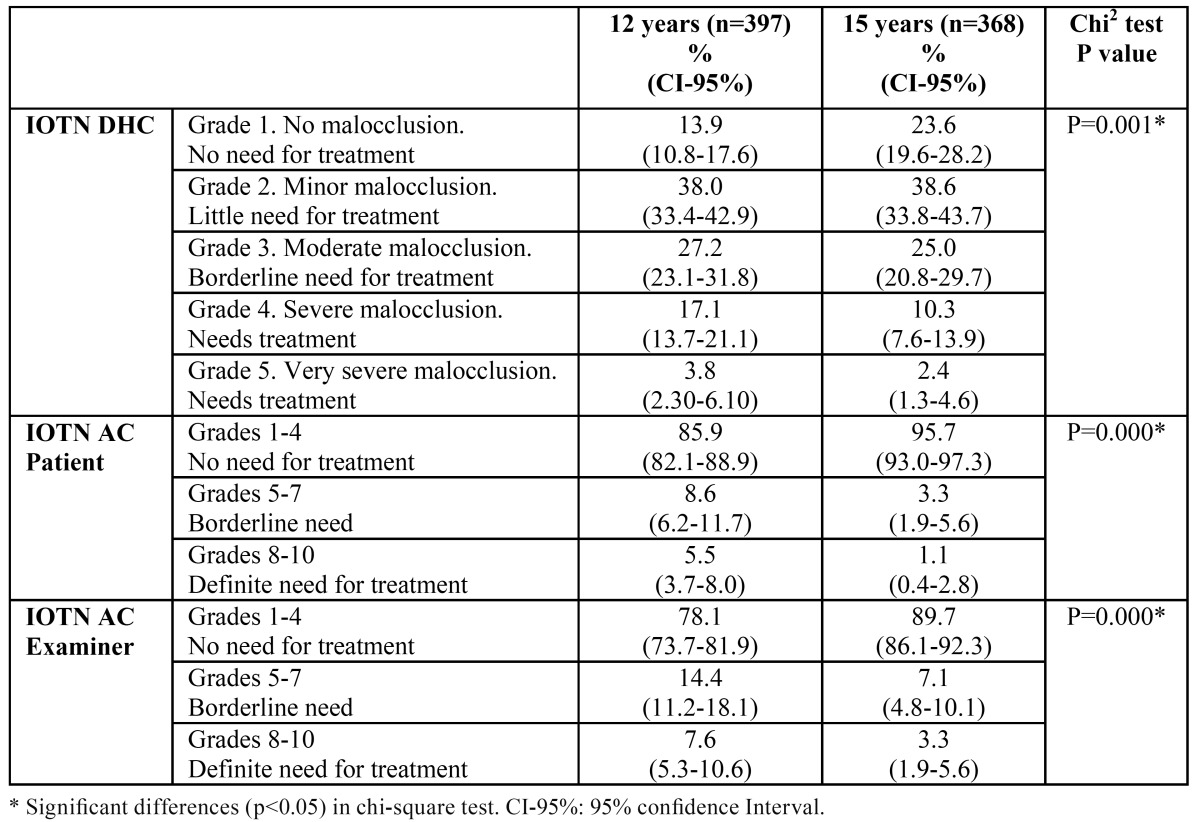
Percentage distribution of Index of Orthodontic Treatment Need (IOTN) grades at 12 and 15 years of age.

The diagnostic agreement between the DAI and the modified IOTN was moderate, obtaining Kappa scores of 0.426 for the 12-year-old group and 0.415 for the 15-year-old group.

On comparing the orthodontic treatment need after excluding those who had received orthodontic treatment (n=637), no signifi-cant differences were found in either the DAI (p=0.07) or the IOTN (p=0.31) results.

In relation to gender, no significant differences were found with any of the indices employed, whether DAI (chi-square test p=0.414), IOTN DHC (chi-square test p=0.186), IOTN AC patient (chi-square test p=0.095), IOTN AC examiner (chi-square test p=0.075) or modified IOTN (chi-square test p=0.139).

 Table 3 presents the percentage distribution of the different grades of treatment by index and social class. No significant differences were found with the DAI (chi-square test p=0.533), IOTN DHC (chi-square test p=0.062) or IOTN AC assessed by the children (chi-square test p=0.622). However, the IOTN AC assessed by the examiner showed a significantly higher treatment need in the low social class group (chi-square test p=0.009).

**Table 3:**
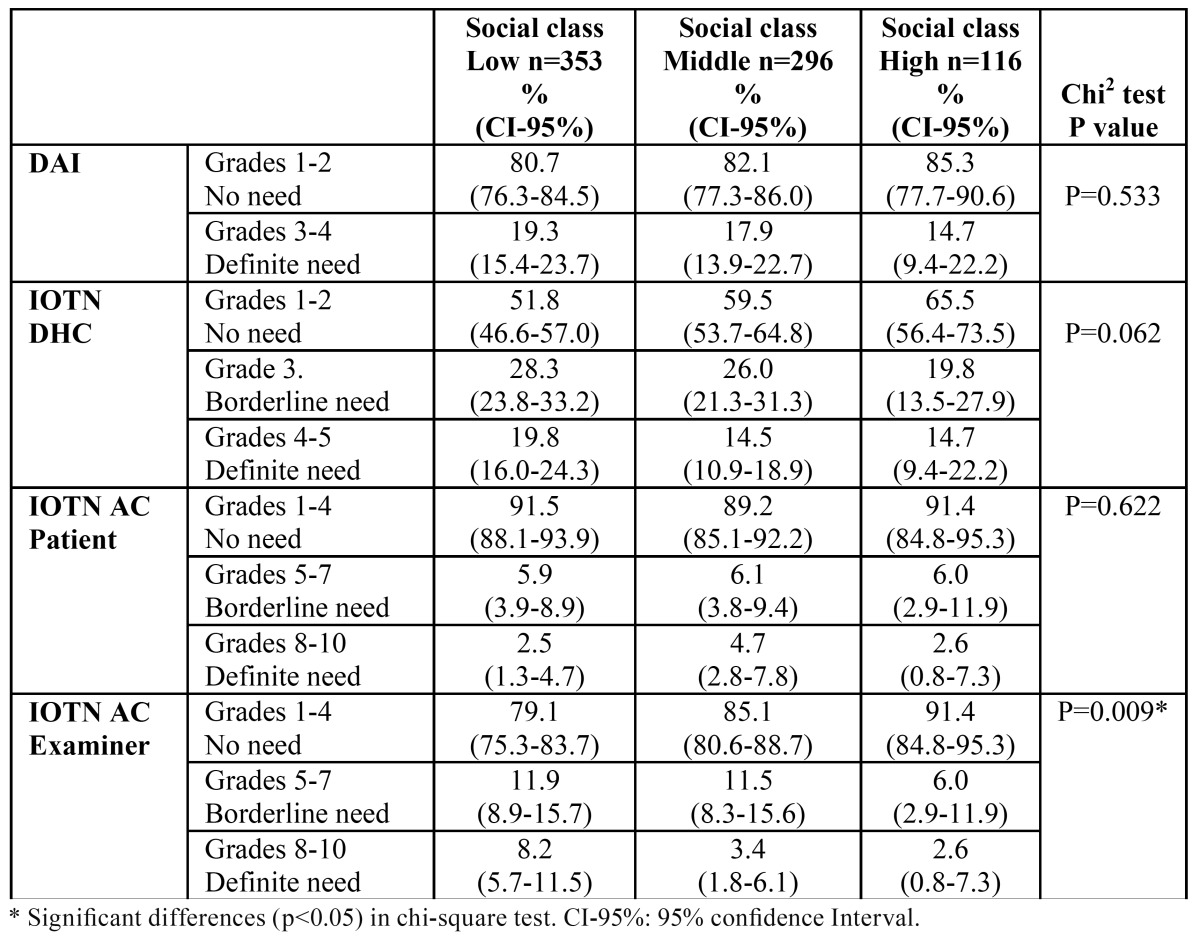
Percentage distribution of the different grades of treatment by index and social class.

## Discussion

Many occlusal indices have been developed to classify malocclusions according to their severity or orthodontic treatment need. Although there is a certain consensus about the requirements that occlusal indices must meet, it is very difficult to develop an index that can win the recognition of the international research community, making it difficult for this work to develop and hindering subsequent comparisons with other studies.

Whether this type of study should include or exclude persons who have previously received orthodontic treatment has always been a controversial question. Some authors exclude them (5,6) while others include them (7-9). In order to obtain a representative sample, patients undergoing orthodontic treatment at the time the examinations took place were excluded from the present study but those who had received such treatment in the past were included.

On examining the orthodontic treatment need by age group, it was observed that the 15-year-olds had significantly less need of treatment than the 12-year-olds. This result was observed irrespectively of the index employed, whether DAI, IOTN DHC, patient-assessed IOTN AC or examiner-assessed IOTN AC. It is explained by the percentage who had previously received ortho-dontic treatment, which was significantly higher in the 15-year-old group than in the 12-year-old group. Moreover, the differences in orthodontic treatment need ceased to be statistically significant when those who had received orthodontic treatment in the past were excluded.

The orthodontic treatment need assessed by the DAI was 21.7% at 12 years of age and 14.1% at 15 years. Manzanera et al. obtained very similar treatment needs: 21.2% and 16.1% for the 12 and 15-year-old groups respectively (10). Other studies have also encountered very similar figures (11-14).

The orthodontic treatment need assessed by the IOTN DHC was 20.9% at 12 years of age and 12.7% at 15 years. Manzanera et al. obtained very similar results (21.8% and 17.1% respectively) in a representative sample from the same region (3). In France, Souames et al. found 21% in a sample of children aged 9 to 12 years (14). In Iran, Hedayati et al. obtained similar results (18.4%) in a sample aged between 11 and 14 years, as did Puertes-Fernández et al. in a sample of 12-year-old children in the Western Sahara (18.1%) (15,16).

The IOTN AC assessed by the children gave treatment needs of 5.5% at 12 years and 1.1% at 15 years. Similar results have been found in other studies such as Manzanera et al., Souames et al., Hedayati et al., Josefsson et al., Nobile et al. and Hamdan, alt-hough Abdullah and Rock obtained a far higher percentage (3,14,15,17-20).

It should not be forgotten that although the IOTN AC is widely employed to ascertain the patients’ own perceptions of their malocclusions, its validity has been questioned by some authors (21-23).

It was also found, as in other studies that the treatment need assessed by the patients (AC patient) was lower than that assessed by the examiner (AC examiner) (24,25).

In relation to gender, no significant differences were found with any of the indices employed, which is in agreement with other authors (3).

The treatment need assessed by the DAI and the IOTN was slightly higher in the low social class than in the middle and high classes. While these differences were not significant, they nonetheless indicate a trend that has been displayed more strongly in other studies (2,26).

The low diagnostic agreement between the different treatment need indices was borne out in the present study by the moderate kappa figure for agreement between DAI and modified IOTN, similar to that obtained by other authors (27,28).

It may be stated that approximately 20% of the children needed orthodontic treatment and neither gender nor social class appeared to exert a significant influence on orthodontic treatment need.
